# EAE models of neuropathic pain in multiple sclerosis do not require pertussis toxin

**DOI:** 10.1016/j.brainres.2026.150162

**Published:** 2026-01-11

**Authors:** Sydney R. Lamerand, Paramita Basu, Nina E. Gakii, Skyy S. Steber, Bradley K. Taylor

**Affiliations:** a Department of Anesthesiology and Perioperative Medicine, Department of Pharmacology, Pittsburgh Center for Pain Research, and Pittsburgh Project to end Opioid Misuse, University of Pittsburgh School of Medicine, Pittsburgh, PA, USA; b Center for Neurosciences at the University of Pittsburgh, Pittsburgh, PA, USA

**Keywords:** Multiple sclerosis, Experimental autoimmune encephalomyelitis, Pertussis toxin, Allodynia, Hyperalgesia, Hypersensitivity, Pain

## Abstract

**Background:**

Experimental autoimmune encephalomyelitis (EAE) is a preclinical model of multiple sclerosis (MS), typically induced with two inoculations of myelin oligodendrocyte glycoprotein (MOG_35–55_) emulsified in complete Freund’s adjuvant (CFA), and supplemented with pertussis toxin (PTX). Although PTX has been considered essential, recent studies suggest that EAE pathology can develop without it.

**Objectives:**

Indices of clinical disease and neuropathic pain were evaluated in a conventional model of EAE that included PTX (EAE-PTX) and one that lacked PTX (EAE-nPTX), as well as in multiple control groups that lacked MOG_35–55_ (CFA-PTX and CFA-nPTX).

**Methods:**

A battery of behavioral tests were used to evaluate motor dysfunction and hypersensitivity to mechanical, cold, and heat stimuli with a repeated-measures design in male and female C57BL/6 mice. One month after the first EAE inoculation, fluoromyelin staining was used to evaluate demyelination in spinal cord, cortex, and peripheral nerve, while ATF3 was used as a marker of injury in sensory neurons of lumbar L4-L5 dorsal root ganglia (DRG).

**Results:**

Compared to CFA-PTX and CFA-nPTX controls, both EAE-PTX and EAE-nPTX groups developed motor dysfunction, behavioral hypersensitivity, and demyelination in ventral spinal cord but not cortex. Spinal demyelination was greater in EAE-nPTX than in EAE-PTX. ATF3 was detected in lumbar DRG of all EAE and CFA control groups, suggesting that systemic inflammation, rather than MOG_35–55_-driven neuropathology, contributes to neuron damage.

**Conclusions:**

PTX is not required for the manifestation of motor dysfunction and neuropathic pain in MOG_35–55_-based EAE models. Newer EAE-nPTX models have the distinct advantage of mimicking MS disease while avoiding confounding effects of pertussis toxin.

## 1. Introduction

Multiple sclerosis (MS) is an autoimmune neurodegenerative disease of the central nervous system (CNS) that presents as cognitive impairment (Benedict et al., 2013; Bisecco et al., 2015), walking and gait abnormalities associated with corticospinal damage (Morozumi et al., 2024), central neuropathic pain associated with spinothalamic-thalamocortical pathway demyelination (Rivel et al., 2021), and vision impairment associated with optic nerve demyelination (Costello, 2016). Key features of MS including neuroinflammation, demyelination, neurodegeneration, paralysis, and pain are commonly recapitulated in a murine model of experimental autoimmune encephalomyelitis (EAE) (Constantinescu et al., 2011; Iannitti et al., 2014; Yu et al., 2020). EAE is usually induced with two subcutaneous inoculations of myelin oligodendrocyte glycoprotein peptide (MOG_35–55_), emulsified in complete Freund’s adjuvant (CFA) and supplemented with pertussis toxin (PTX) (Bjelobaba et al., 2018; Delarasse et al., 2013; Robinson et al., 2014). MOG_35–55_ primes autoreactive T cells in peripheral lymphoid tissues, enabling them to cross the blood–brain-barrier (BBB) in the setting of CFA-induced immune system activation and disruption of the BBB (Fletcher et al., 2010; Furtado et al., 2008). Supplemental PTX is thought to enhance immune cell infiltration into the CNS by further disrupting BBB integrity (Linthicum, 1982). Although EAE serves as a valuable model, it does not fully recapitulate the complexities of MS. For example, while most studies can demonstrate spinal demyelination in EAE, the pathology of MS is characterized by cortical and cerebellar demyelination as well (Constantinescu et al., 2011; Gran et al., 2008; Sriram & Steiner, 2005). Indeed, brain demyelination is a key trait of clinical MS that is rarely replicated in EAE models However, two reports indicate that removal of PTX from the EAE induction paradigm allows for the manifestation of cortical demyelination (Blankenhorn et al., 2000; Murphy et al., 2020), leading some to suggest that PTX might somehow inhibit brain demyelination.

In 1933, the first EAE-like model was developed in non-human primates with 30–100 intramuscular injections of rabbit brain emulsions (Rivers et al., 1933). The model was refined to include Freund’s adjuvant containing a brain emulsion with killed *tubercle bacilli* in 1947 and then simplified to a single injection protocol (Kabat et al., 1947; Kabat et al., 1951). In the 1950 s, rat and guinea pig models of EAE were developed with the use of heat inactivated Mycobacterium tuberculosis in Freund’s adjuvant (i.e. CFA) to enhance CNS sensitization to myelin proteins (Gold et al., 2006). Early EAE models in mice were plagued with inconsistent disease incidence and course (Gold et al., 2006), until PTX was added to MOG and CFA to increase BBB permeability (Linthicum, 1982), and found to increase disease and pain severity in a concentration-dependent (Bernard & Carnegie, 1975; Segal et al., 2020). The requirement of PTX was supported in C57BL/6 mice by Wang et al. (2017), who reported that PTX was necessary for manifestations of motor dysfunction and demyelination (Wang et al., 2017). However, these studies did not attempt to compensate for the absence of PTX with the addition of a second, booster injection to promote disease development. When this was attempted using a PTX-free, double immunization protocol, two studies reported hindlimb paralysis in either BC1 (Blankenhorn et al., 2000) or C57BL/6 mice (Murphy et al., 2020). However, these latter studies failed to include a PTX-inclusion comparison group and so did not directly compare groups with or without the inclusion of PTX in the induction cocktail. To fill this gap, we conducted this side-by-side comparison in a rigorous, well-controlled manner. We compared key pathological features of EAE across a conventional PTX-based EAE model routinely used in our laboratory (EAE-PTX) (Doolen et al., 2018; Iannitti et al., 2014; Rahn et al., 2014) with that of a PTX-free model (EAE-nPTX) (Murphy et al., 2020; Nguyen et al., 2024). To control for variations in adjuvant, we generated a third variant model (vEAE) that was PTX-free but contained the same amount of MOG and CFA as in the EAE-PTX model.

Neuropathic pain is experienced by up to 86 % of MS patients. Conventional EAE-PTX models are classically used to study the hypersensitivity associated with MS (Doolen et al., 2018; Lu et al., 2012; Olechowski et al., 2009). To determine whether PTX is necessary not only for motor dysfunction but also for EAE-associated hypersensitivity to mechanical and cold stimulation, we evaluated paw withdrawal responses to the plantar application of light touch or evaporative acetone (Doolen et al., 2018; Iannitti et al., 2014; Lu et al., 2012; Rahn et al., 2014) in the EAE-PTX, EAE-nPTX, and vEAE models. We and others reported that complete paralysis can be avoided with optimized concentrations of MOG_35–55_, CFA, and PTX. This enabled assessment of paw-withdrawal reflexes for many weeks or months (Doolen et al., 2018; Olechowski et al., 2009; Rahn et al., 2014). With this approach, we determined in a rigorous manner and with necessary control groups whether PTX is necessary for the full manifestation of neuropathic pain in EAE models of MS.

## 2. Methods

### 2.1. Animals

Wild-type male and female C57BL/6 mice were purchased from Jackson Laboratories (Bar Harbor, ME, USA) and arrived 9–11 weeks of age. Mice were housed in a temperature- (20–22 °C) and humidity- (45 ± 10 %) controlled environment on a 12/12 h light/dark cycle, with food and water provided *ad libitum*. All animal procedures were approved by the Institutional Animal Care and Use Committee at the University of Pittsburgh and conducted in accordance with institutional relevant guidelines and regulations, as well as the NIH Guide for the Care and Use of Laboratory Animals. Animals were assigned to one of six groups as shown in Table 1.

### 2.2. Mouse models of EAE and MSNP

#### 2.2.1. Conventional mouse model (EAE-PTX)

Similar to our previous reports (Doolen et al., 2018; Rahn et al., 2014), EAE was induced with MOG_35–55_ (Bio-Synthesis, Lewisville, TX, USA), complete Freund’s adjuvant (CFA, Sigma-Aldrich, St. Louis, MO, USA), and PTX (List Labs, Campbell, CA, USA). CFA was prepared with the addition of heat-inactivated *Mycobacterium tuberculosis* H37Ra (MTB; Fisher Scientific, Waltham, MA, USA; 3 mg/ml) to incomplete Freund’s adjuvant (IFA; Sigma-Aldrich, St. Louis, MO, USA). MOG_35–55_ (150 μg, s.c.) powder was emulsified in a 1:1 solution of filtered 1x phosphate-buffered saline (PBS; Fisher Scientific, Waltham, MA, USA) and CFA. MOG + CFA was bilaterally injected (100 μl) at the flank of each hindlimb under gentle manual restraint. Immediately following MOG + CFA, PTX (List Labs, Campbell, CA, USA) was administered (200 ng/200 μl, i.p.) on Day 0 (D0). PTX was injected again on D2 at the same dose. The MOG_35–55_/CFA injection was repeated on Day 6 (D6) for a total amount per mouse of 300 μg MOG_35–55_, 200 μL CFA (3 mg/ml MTB), and 200 μL PBS (400ul total injection volume across both hind flanks and both injection days) plus 400 ηg PTX total (i.p.). Controls received identical injections in the absence of MOG35–55 (deemed CFA-PTX Controls).

#### 2.2.2. Mouse model with no PTX (EAE-nPTX)

We administered MOG_35–55_ (Bio-Synthesis, Lewisville, TX, USA) and complete Freund’s adjuvant (CFA) with no pertussis toxin to induce an EAE model of MS (Murphy et al., 2020; Wang et al., 2017). Complete Freund’s adjuvant (CFA; InvivoGen, San Diego, CA, USA) containing heat-inactivated *Mycobacterium tuberculosis* H37Ra (MTB; 1 mg/mL) supplemented with additional MTB (4 mg MTB/ml CFA; Fisher Scientific, Waltham, MA, USA) for a final concentration of 5 mg/mL MTB/CFA. MOG_35–55_ was dissolved in PBS and combined in a 1:1 solution with CFA. The MOG + CFA + PBS emulsion was prepared using a Tissue Tearor (Biospec Products, Bartlesville, OK, USA) for 1 min on speed setting 6 and passed through 1 ml syringes 1–3 times as needed to remove any air bubbles. Mice were injected in each hindflank (50 μL/hindflank, subcutaneously, s.c.) using a 20-gauge needle attached to the 1 mL syringe of MOG + CFA + PBS emulsion on Day 0 (D0) with the following volumes per hindflank: MOG_35–55_ (50 μg), MTB/CFA Controls received identical injections in the absence of MOG35–55 (deemed CFA-nPTX Controls).

#### 2.2.3. vEAE mouse model with no PTX and with MOG, MTB, and CFA varied to match EAE-PTX

Any difference in outcome measures between the EAE-PTX and EAE-nPTX models could be due to either PTX or due to variations in the amount of MOG, IFA, MTB, or MOG in the induction cocktail composition (Table 1). To account for the latter, we added a third control group without PTX, termed the vEAE group, that received the other components that were varied to match those described for the EAE-PTX model. To establish a sham control for the vEAE group, termed the vCFA group, MOG was omitted.

### 2.3. Behavioral assessment of motor and sensory functions

An investigator blinded to treatment groups conducted behavioral testing, starting as early as 4 days before the first MOG/CFA injection (D0). An assistant assigned treatment groups in a balanced manner to minimize baseline threshold variability.

#### 2.3.1. Neuromotor Dysfunction

Signs of motor dysfunction were scored according to the following clinical assessment scale (Murphy et al., 2020): 0 – no motor deficits, 1 – 25 % tail flaccidity, 1.5 – 50 % tail flaccidity, 2 – full tail flaccidity, 2.5 – unsteady gait (slight wobble), 3 – bilateral paresis (hind paws drag before plantar step), 3.5 – full hindlimb paralysis, 4 – forelimb paralysis.

Mice that developed a motor score of 3.5 or higher (indicative of full hindlimb paralysis) were excluded from behavioral analysis. Less than 5 % of animals exceeded this threshold. Mice with scores up to 3.0 (hindlimb paresis) were included, as they retained sufficient hindlimb mobility to exhibit reliable withdrawal responses to mechanical and thermal stimuli. This criterion ensured that motor deficits did not confound pain sensitivity assessments.

#### 2.3.2. Mechanical sensitivity

As previously described (Basu et al., 2021), mice were acclimated to a temperature- and light-controlled room in individual Plexiglas boxes placed on top of a stainless-steel mesh platform for 45 min prior to behavioral testing (2 days of habituation plus testing day(s)). Mechanical thresholds in response to a non-noxious mechanical stimulus were determined using an incremental series of 8 von Frey filaments (Stoelting) of logarithmic stiffness (0.008–6 g). Filaments were applied perpendicular to the skin surface, directly to the center of the left plantar hind paw, with enough force to allow for a slight bending of the filament. When the left hind paw was unable to be tested due to a clinical score of 3.5, the right hindpaw was assessed. Positive response was defined as a rapid withdrawal of the hind paw within 4 s. Gram force was logarithmically converted, and 50 % mechanical withdrawal threshold was determined using an up-down method (Chaplan et al., 1994).

#### 2.3.3. Cold Sensitivity

Immediately following von Frey testing, mice were assessed in the same acrylic chambers for response duration to evaporative coolness as previously described (Qi et al., 2023). An acetone droplet was applied to the center of the hind paw plantar skin using a syringe connected to a PE-90 tubing applicator with a flared tip, 3.5 mm in diameter. The volume of the droplet maintained on the top of the applicator ranged from 10 to 12 μl. The droplet was applied with care to avoid contact between the skin and applicator. Mice were observed for 60 s following acetone application. The duration of time the animal lifted, shook, or licked its paw was recorded. A total of 3 trials, applied at 1 min intervals, were averaged.

#### 2.3.4. Heat Sensitivity

Heat sensitivity was tested with a radiant heat paw-withdrawal assay (Ugo Basile, Italy) (Hargreaves et al., 1988). Mice were placed in clear Plexiglas boxes on an elevated glass platform and allowed to acclimate for 30 min prior to testing. The radiant heat source was positioned under the glass platform directly beneath the hind paw and thermal stimulus was applied. If the mouse did not respond within 20 s, heat stimulation was discontinued to prevent tissue damage to the hind paw. Withdrawal responses were measured three times, at 5 min intervals, and response latencies were averaged.

### 2.4. Tissue preparation

Animals were deeply anesthetized with pentobarbital (Fatal Plus, diluted to 200 mg/kg i.p., Med-Vet International, Mettawa, IL) and perfused transcardially with 20 ml of room temperature (RT), 0.01 M phosphate buffered saline (PBS) with heparin (10,000 USP units/L) followed by 20 ml of ice-cold fixative (10 % buffered formalin). Lumbar spinal cords, brains, sciatic nerves and lumbar region L4-L5 DRGs were removed and post-fixed overnight in 10 % buffered formalin (4 °C) and then cryoprotected for 36–96 hr in 30 % sucrose in 0.01 M PBS. Spinal L4-L6 transverse sections (30 μm) were cut on a sliding microtome with freezing stage and collected in 0.01 M PBS. They were either mounted on microscope slides and allowed to dry before storing at −20 °C, or left free floating for IBA-1 staining. Coronal brain, sciatic nerve and DRG sections were cut 20um on a cryostat (Cryostar NX70, Fisher Scientific, USA), mounted directly on microscope slides and stored at −20 °C until staining.

### 2.5. Immunohistochemistry

Mounted or free floating sections were washed three times in 0.01 M PBS (Fisher Scientific) and then pretreated with 3 % normal goat or donkey serum (Equitech Bio Inc) and 0.3 % Triton X-100 (VWR) to block non-specific binding. Sections were then incubated in a primary antibody for ATF3 (Rabbit anti-ATF3, 1:500, Abcam), CGRP (mouse anti-CGRP, 1:500, Abcam), or IBA-1 (goat anti-IBA1, 1:1000, Sigma-Aldrich) overnight at RT on a slow rocker. The tissue was then washed in 0.01 M PBS 3×10 min and incubated at RT in secondary antibody (Alexa 568-conjugated donkey anti-rabbit, Alexa 488-conjugated goat anti-mouse, or Alexa 568-conjugated donkey anti-goat) at 1:1000 dilution for two hrs. Tissue was washed three times in 0.01 M PBS for 10 min, twice in 0.01 M PB for 10 min and once in distilled water for 5 min. Floating sections for IBA-1 were mounted and dried at RT before they were cover-slipped with Vectashield hardset antifade mounting medium with DAPI (Vector Laboratories), dried at RT for 30 min and then stored at 4 °C until imaging.

### 2.6. Fluoromyelin staining

To assess demyelination, mounted sections were washed 3×10 min in 0.01 M PBS and then incubated for 30 min in Green Fluorescent Myelin Statin (FluoroMyelin^™^, ThermoFisher, 1:300). We chose to use FluoroMyelin instead of another lipid-associated myelin staining method, Luxol Fast Blue, because it yields comparable results while negating the need for paraffin embedding (Kanaan et al., 2006; Pellicciari et al.). Tissue was then washed twice in 0.01 M PBS for 10 min, once with distilled water for 5 min, dried at RT, cover-slipped with DAPI, and then stored at 4 °C until imaging.

### 2.7. Fluorescence microscopy and image processing

Images of sections were captured on a Nikon Ti2 inverted epifluorescence microscope equipped with a motorized stage and a Prime BSI camera (Photometrics, USA). Within each experiment, the same exposure time was used for all images captured within each channel. Stitched images were taken with a 10 % overlap around an area of interest at 10X, 20X, or 40X magnification using ND acquisition software. Image capture, adjustments, and quantification were performed in NIS Elements (V 5.30.06 Nikon, Japan). An investigator blinded to treatment group first adjusted LUTs intensities in the same manner for each image following image acquisition. For each animal, 5 sections in each region of interest (i.e. corpus callosum, L4-L5 spinal cord, L4-L5 DRGs, and sciatic nerve) were selected for quantification based on section quality. For spinal cords, an ROI was drawn in the ventral spinal cord white matter. For brains an ROI was drawn around the corpus callosum. For sciatic nerves an ROI was drawn around the entire nerve. Where staining artifacts or tissue folds were present, that area was excluded from the ROI. Average fluoromyelin intensity of each ROI was determined with NIS-Elements GA3 software. The changes in intensity values correspond to changes in myelin content. Average intensity across sections within each group were averaged to yield one data point per animal per tissue type. For analysis of ATF3 staining, ATF3-positive cells were counted manually in each lumbar DRG section. Four to five sections per animal were analyzed, and the average number of positive neurons per section by area was calculated for each group. A naïve control group was included to determine ATF3 expression in untreated mice.

### 2.8. Statistical analysis

Data were graphed and analyzed with Prism software (GraphPad 10, San Diego, CA, USA). All data are expressed as mean ± SEM and statistical significance was set at P < 0.05. Behavioral data plotted over time were analyzed by two-way ANOVA (α = 0.05) with Model as the between-subjects factor and Time as the repeated measure. Data were also plotted as the average across Day 8 – Day 28 by calculating area under the curve (AUC). AUC data was analyzed with one-way ANOVA (α = 0.05) with Model as the between-subjects factor for all 6 groups. Histochemical intensity data was analyzed by 2-way ANOVA with Model (EAE-PTX, EAE-nPTX, and vEAE) and Group (MOG included, MOG not included) as between subjects factors. Tukey’s test for post hoc comparisons were conducted when appropriate.

## 3. Results

### 3.1. 3.1.EAE-PTX and EAE-nPTX mice exhibit comparable motor dysfunction and hypersensitivity

**Fig. 1.**
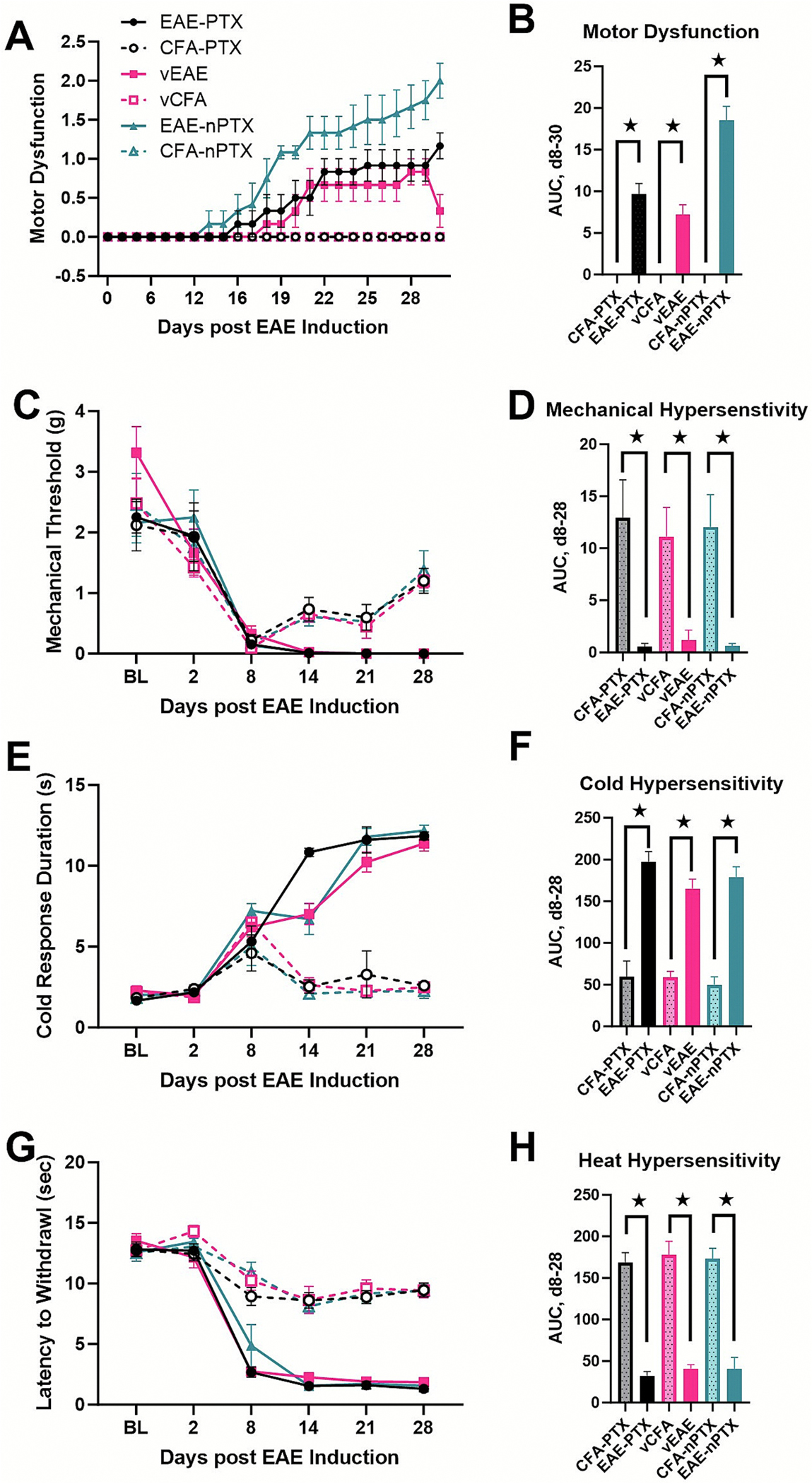
Motor dysfunction and plantar hypersensitivity develop in EAE-nPTX mice. (A,B) Development of motor dysfunction in EAE groups with inclusion in the induction cocktail of CFA and PTX (EAE-PTX), CFA but not PTX (EAE-nPTX), and variant concentrations of MOG, CFA and PTX (vEAE). Development of (C,D) mechanical hypersensitivity, (E,F) cold hypersensitivity, and (G,H) heat hypersensitivity the three EAE groups. CFA controls developed signs of (C,D) mechanical hypersensitivity that was significantly less than EAE by D14–28. CFA controls developed (E,F) cold hypersensitivity, and (G,H) heat hypersensitivity on D8 but significantly different from EAE by D14 AUC = Area under the curve. n = 6 per group. Data represent mean ± SEM). ★ EAE group significantly different from respective CFA control.

As illustrated in Fig. 1, behavioral signs of motor dysfunction and hypersensitivity were observed in both EAE-PTX mice as previously reported (Doolen et al., 2018; Iannitti et al., 2014; Lu et al., 2012; Rahn et al., 2014), in EAE-nPTX mice as previously reported (Murphy et al., 2020; Nguyen et al., 2024), and in vEAE mice.

Interestingly, motor dysfunction in EAE-nPTX mice developed more quickly and to a greater degree than in EAE-PTX and vEAE mice (Fig. 1A-B). While motor dysfunction persisted throughout the 30-day study in EAE-PTX and EAE-nPTX groups, this began to resolve by D30 in the vEAE group. As expected, none of the CFA-only (no MOG) controls developed motor dysfunction.

All three EAE groups developed comparable signs of mechanical (Fig. 1C-D) and cold (Fig. 1E-F) and heat (Figure G-H) hypersensitivity, beginning on D8 and persisting through D30. As in previous studies, CFA-only controls groups exhibited transient mechanical and thermal hypersensitivity that peaked at D8. These signs of acute peripheral inflammation quickly resolved and were always less than in their corresponding MOG-containing groups.

We performed a post hoc analysis of motor dysfunction and hypersensitivity stratified by sex (Supplementary Fig. 1). Males in the EAE-PTX and vEAE groups exhibited greater motor dysfunction than their female counterparts, while males in the EAE-nPTX group showed lesser motor dysfunction than females. In both sexes, motor dysfunction in the EAE-nPTX group was greater than in the other groups, consistent with a more severe phenotype. With respect to behavioral signs of neuropathic pain, mechanical and cold hypersensitivity was similar between males and females. Heat hypersensitivity in males and females was similar in the EAE-PTX and vEAE groups and their CFA control groups, but slightly higher in males in the EAE-nPTX group.

### 3.2. EAE-PTX and EAE-nPTX mice exhibit comparable demyelination in ventral spinal cord

**Fig. 2.**
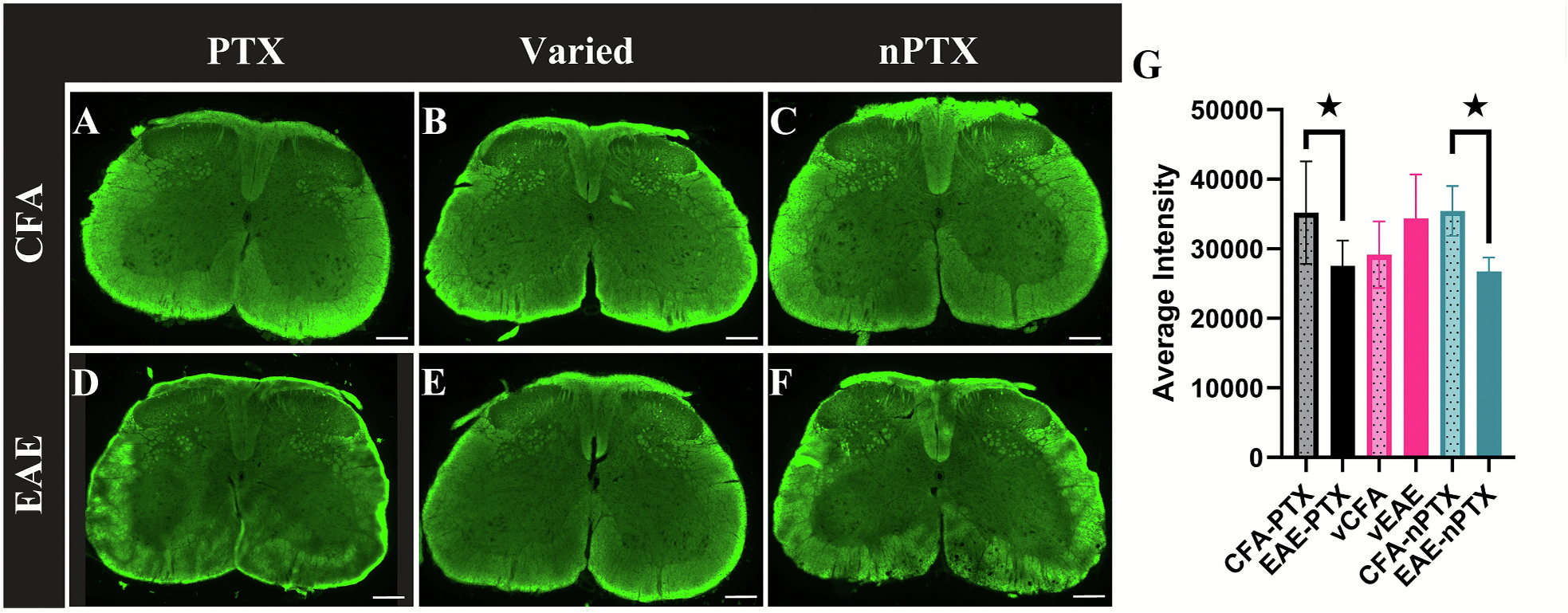
EAE-nPTX mice exhibit demyelination in ventral spinal cord. Representative images illustrating fluoromyelin staining in lumbar spinal cord, 30 days after administration in control groups not containing MOG (A) CFA-PTX, (B) vCFA, (C) CFA-nPTX; and in EAE groups with MOG present, (D) EAE-PTX, (E) vEAE, (F) EAE-nPTX. (G) Mean intensity of Fluoromyelin staining in ventral horn. n = 6 per group. Values represent mean ± SEM. Scale bar: 250 μm. ★ indicates p < 0.05 in EAE group compared to respective CFA control group.

A defining feature of both clinical MS and preclinical EAE models is demyelination in the ventral horn of the lumbar spinal cord (Mey et al., 2022; Murphy et al., 2020; Ransohoff, 2012; Schellenberg et al., 2007; Wu et al., 2008). As expected, the EAE-PTX group exhibited patches of demyelination (Fig. 2A–F), and FluoroMyelin staining intensity was reduced by 21.7 % as compared to their CFA control (p < 0.05, Fig. 2G). Similarly, FluoroMyelin staining intensity was reduced by 24.5 % in the EAE-nPTX group as compared to their CFA control (p < 0.05), suggesting that PTX is not necessary for spinal cord demyelination. Interestingly, the vEAE model exhibited an increase (17 %), rather than a decrease, in FluoroMyelin staining intensity compared with its CFA control, pointing to a greater importance of MOG and CFA concentration (relative to presence or absence of PTX) in the presentation of demyelination in EAE models.

### 3.3. EAE is not associated with cortical demyelination, regardless of PTX

**Fig. 3.**
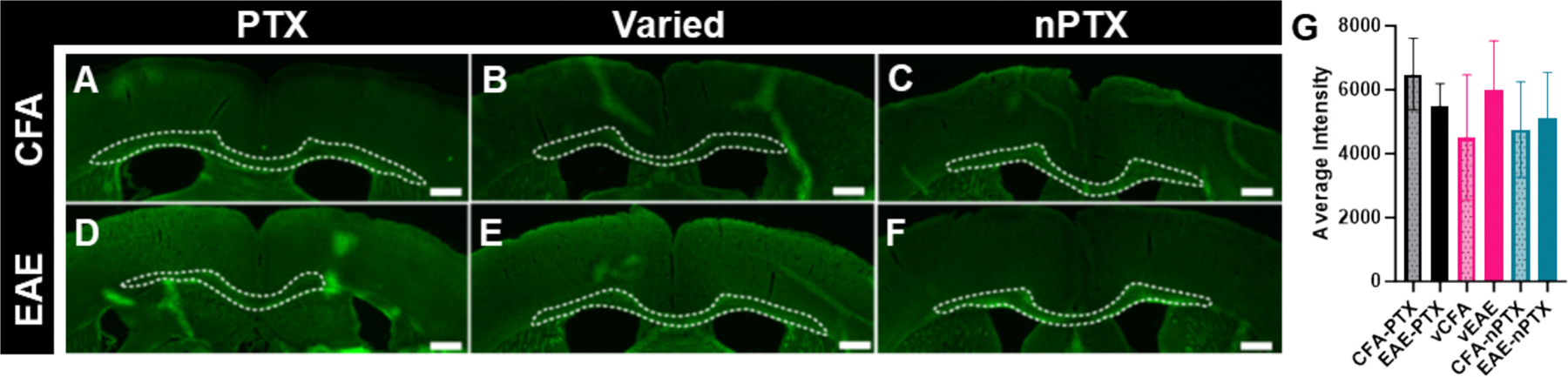
EAE is not associated with cortical demyelination. Representative images illustrating fluoromyelin staining in corpus callosum, 30 days after induction in control groups with MOG absent (A) CFA-PTX, (B) vCFA, (C) CFA-nPTX, and in EAE groups containing MOG, (A) CFA-PTX, (B) vCFA, (C) CFA-nPTX; and in EAE groups with MOG present, (D) EAE-PTX, (E) vEAE (varied), and (F) EAE-nPTX. (G) Average intensity of fluoromyelin staining in corpus callosum (white-dotted line). n = 5–6 per group. Values represent mean ± SEM. Scale bar: 500 μm.

Cortical demyelination and neuronal pathology are defining features of MS (Calabrese et al., 2010; Cawley et al., 2015; Lucchinetti et al., 2011; Potter et al., 2016). MS patients exhibit demyelination in corpus callosum and axonal damage that correlates with cognitive impairment and upper-extremity dysfunction (Ozturk et al., 2010; Rimkus Cde et al., 2011). However, conventional PTX-containing EAE models rarely if ever develop cortical lesions (Lagumersindez-Denis et al., 2017; Orefice et al., 2020). To determine whether the removal of PTX reveals cortical demyelination in EAE-nPTX mice, we evaluated myelination in the corpus callosum with Fluoromyelin Green staining. Although the cortical myelin signal was less intense than in spinal cord sections due to naturally lower myelin density, fluoromyelin could still delineate gray and white matter boundaries. We did not detect focal myelin loss or structural damage in any group (Fig. 3A-F). Fluoromyelin staining was similar across all groups (Fig. 3G).

### 3.4. EAE is not associated with peripheral demyelination, regardless of PTX

**Fig. 4.**
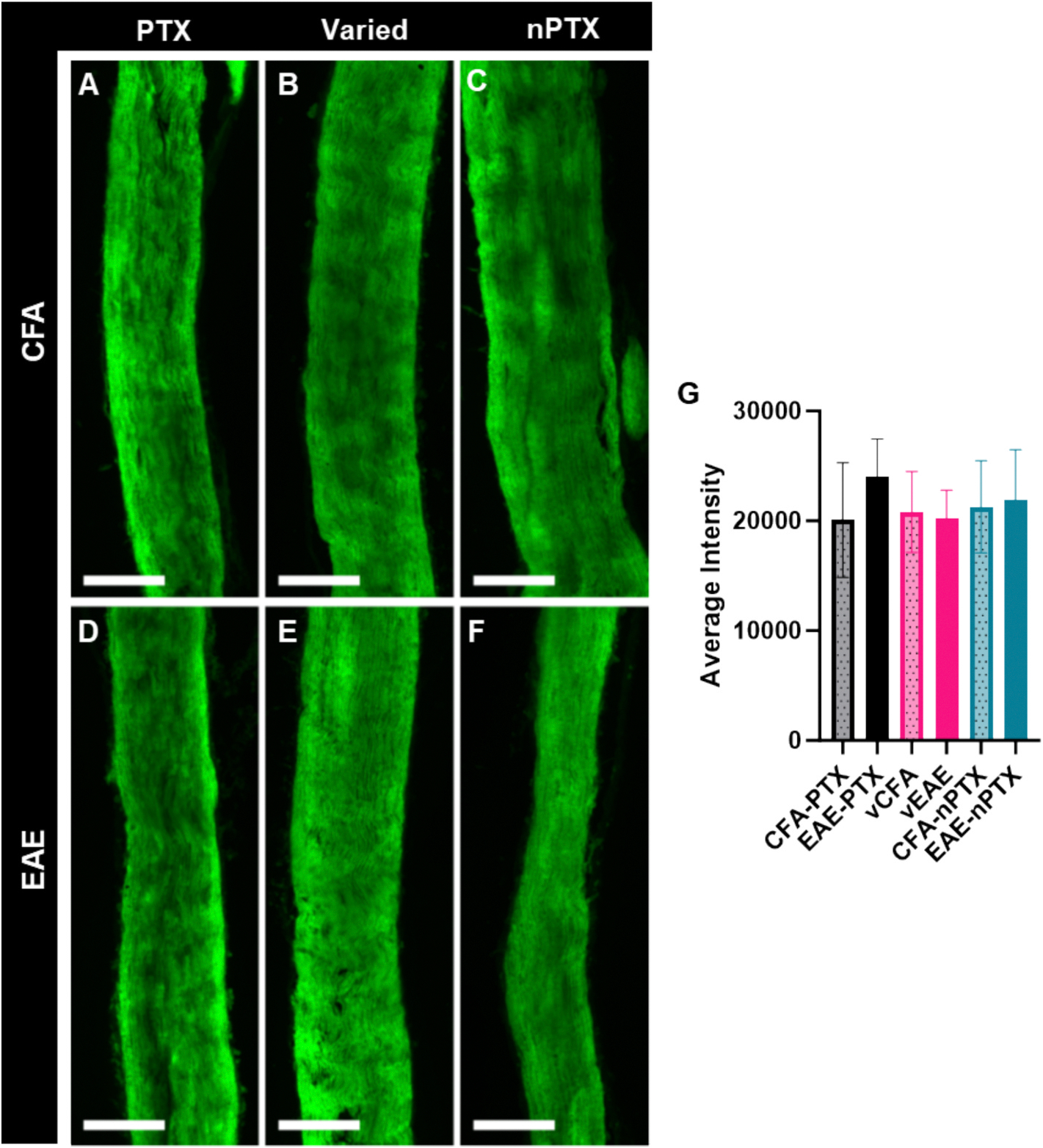
EAE is not associated with peripheral demyelination. Representative images illustrating fluoromyelin staining in the sciatic nerve, 30 days after induction in control groups with MOG absent, (A) CFA-PTX, (B) vCFA, (C) CFA-nPTX; or with MOG present, (D) EAE-PTX, (E) vEAE, (F) EAE-nPTX. (G) Average intensity of fluoromyelin staining in sciatic nerve. n = 3–6 per group. Values represent mean ± SEM. Scale bar: 250 μm.

Neuropathy in multiple sclerosis is traditionally considered to be confined to the CNS, without peripheral demyelination or damage (Rizvi et al., 2021; Suanprasert et al., 2019). However a subset of MS patients (~5%) develop peripheral demyelinating neuropathy (Misawa et al., 2008); therefore, we examined fluoromyelin staining in sciatic nerve. As illustrated in Fig. 4A-F, sciatic nerve myelination was similar in all groups, with no difference in Fluoromyelin staining intensity (Fig. 4G). We conclude that EAE is not associated with peripheral demyelination, regardless of whether PTX is included in the induction cocktail.

### 3.5. CFA upregulates ATF3 in sensory neurons, regardless of the presence of MOG_33–55_ or PTX

**Fig. 5.**
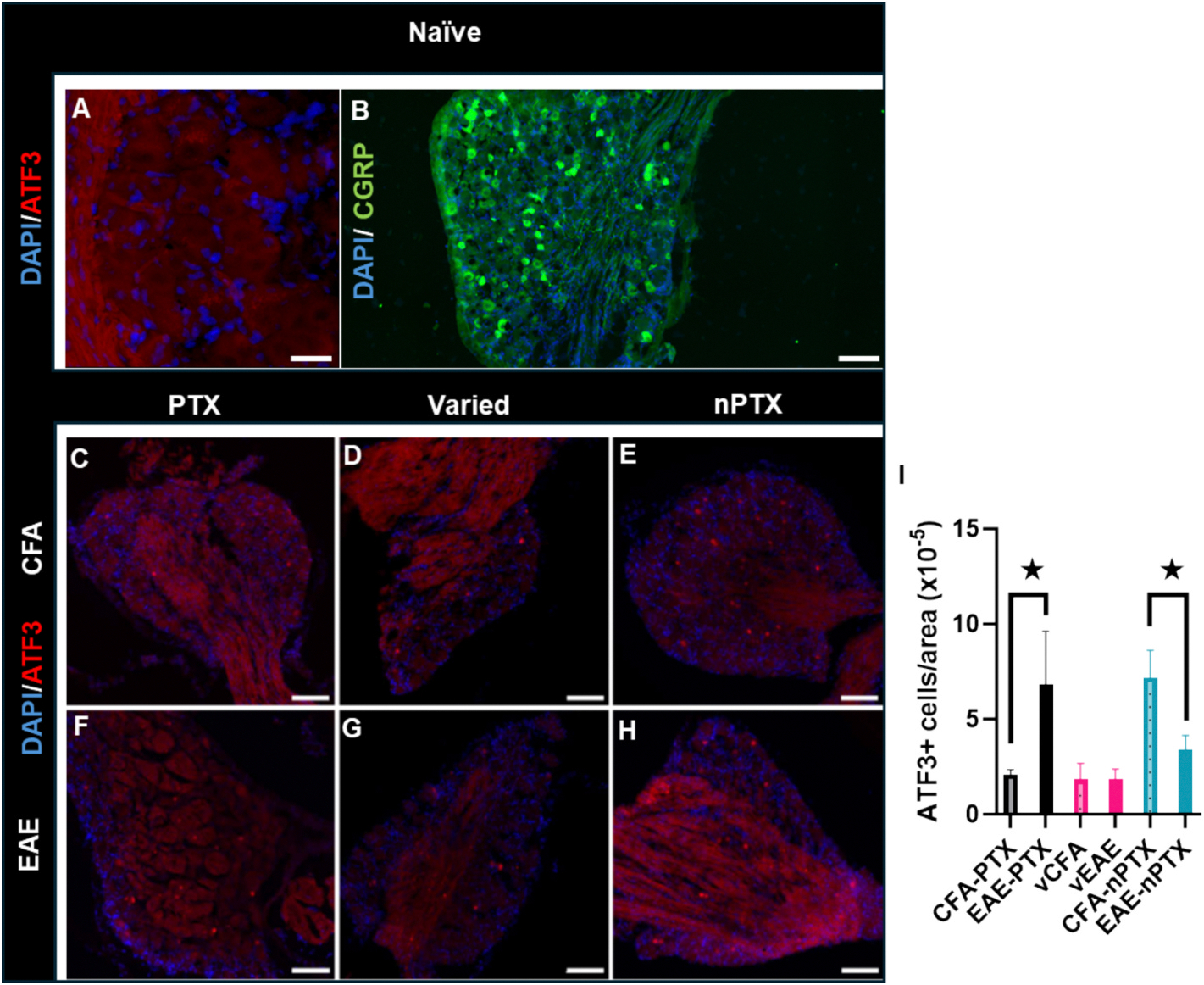
ATF3 expression in lumbar DRG varies across EAE models and controls. Representative images illustrating (A) ATF3 and (B) CGRP staining in the L4 lumbar DRG of naïve mice. Representative images illustrating ATF3 staining, 30 days after induction of (C) CFA-PTX, (D) vCFA, (E) CFA-nPTX, (F) EAE-PTX, (G) vEAE, (H) EAE-nPTX. (I) Quantification of ATF3-positive L4-L5 neurons/DRG area. n = 6 per group. Scale bar: 100 μm. Values represent mean ± SEM. ★ indicates p < 0.05 versus respective control.

AFT3 serves as a marker of peripheral neuron damage (Tsujino et al., 2000). In PTX-containing EAE models, both CFA controls (MOG absent) and EAE mice (MOG present) can upregulate activating transcription factor 3 (ATF3) in DRG sensory neurons (Frezel et al., 2016; Yousuf et al., 2019), regardless of the presence of MOG_33–55_ (Yousuf et al., 2019). To determine whether this can occur in an EAE model lacking PTX, we evaluated ATF3 expression in lumbar L4-L5 DRG. As illustrated in Fig. 5, ATF3 expression was minimal (zero to one positive cell per section) in naïve untreated control mice. ATF3-positive neurons were detected in all other groups. ATF3 expression was greater in EAE-PTX mice as compared to CFA-PTX controls; by contrast, ATF3 expression was less in EAE-nPTX mice as compared to their CFA-nPTX controls. ATF3 expression was similar between vEAE and vCFA control groups. These results confirm and extend the findings of previous studies, suggesting that it is exposure to CFA that causes peripheral neuron damage, and so unrelated to EAE. Our studies also suggest EAE-nPTX models preclude PTX amplification of injury to sensory neurons and thus better represent clinical MS.

### 3.6. Central inflammation in the spinal cord of EAE-nPTX mice

**Fig. 6.**
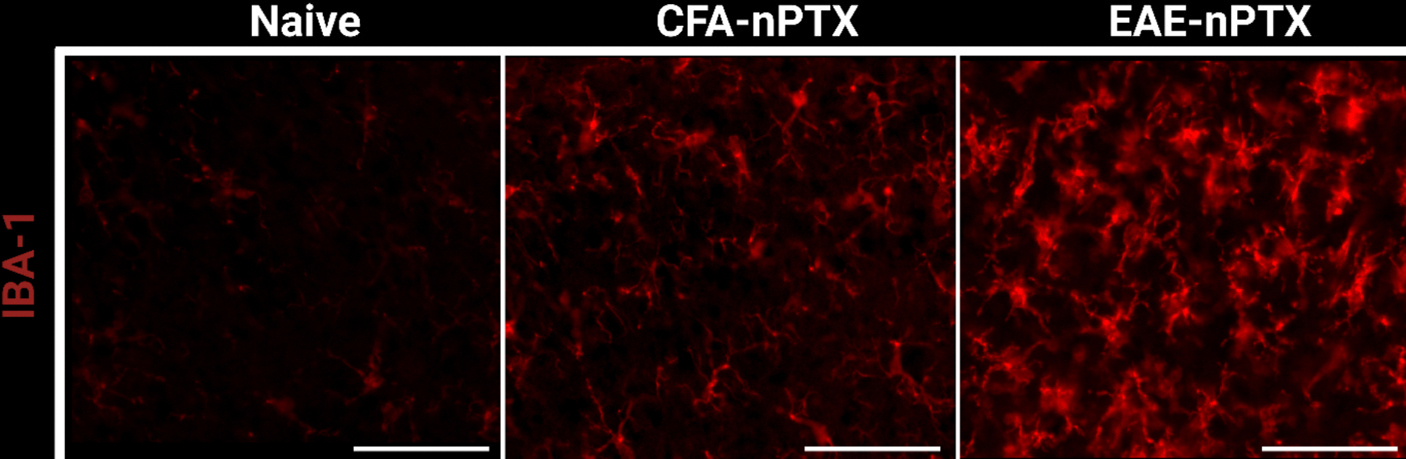
Spinal microglial proliferation in the EAE-nPTX model. Representative Iba1 immunostaining in the dorsal horn of naïve mice (left), mice treated with CFA (CFA-nPTX, middle), and mice treated with MOG and CFA in the absence of PTX (EAE-nPTX, right). Scale bar = 100 μm.

In additional to CNS demyelination, central inflammation including microglial activation is another defining feature of MS patients (Airas et al., 2018; Zrzavy et al., 2017). We and others have describes signs of central inflammation in conventional models of EAE, such as activation and/or up-regulation of microglia (Catuneanu et al., 2019; Doolen et al., 2018; Olechowski et al., 2009), astrocytes (Doolen et al., 2018; Olechowski et al., 2009), and influx of CD3 + T cells (Catuneanu et al., 2019; Olechowski et al., 2009) in the dorsal horn of the spinal cord. To determine whether central neuroinflammation develops in EAE models without PTX, we evaluated Iba1 immunoreactivity in the dorsal horn at the 28 day timepoint after the first innoculation. As illustrated in Fig. 6, EAE-nPTX mice exhibited marked activation of spinal microglia relative to naïve and CFA-nPTX controls. These findings validate the EAE-nPTX model as a relevant platform for the investigation of MS and MSNP.

## 4. Discussion

### 4.1. PTX is not required for the expression of motor dysfunction or neuropathic pain in EAE

MS-associated lesions are associated with demyelination and central inflammation as well as alterations in pain modulatory pathways (Fernandez-de-Las-Penas et al., 2015; Rivel et al., 2021). Basic science researchers who study pathology and pain in EAE models have usually included PTX in MOG_33–55_-based induction cocktails to facilitate immune cell entry into the CNS (Linthicum, 1982). Inclusion of PTX has been assumed to promote full manifestation of hindlimb paralysis, central demyelination and glial activation, as these were absent when PTX was omitted (Arimoto et al., 2000; Ransohoff, 2012; Segal et al., 2020; Wang et al., 2017). However those studies of EAE were limited to a single immunization with MOG (Segal et al., 2020; Wang et al., 2017), as is routine in many laboratory protocols (Blankenhorn et al., 2000). Even when two PTX boosters were given after a single MOG injection, signs of disease were absent (Segal et al., 2020). To test the hypothesis that sufficient dosing of MOG can induce disease in the absence of PTX, we conducted a comprehensive side-by-side comparison of symptomology and pathology in a well-controlled manner using protocols that included two MOG immunizations. We compared a conventional EAE-PTX model as we had used extensively (Doolen et al., 2018; Fu and Taylor, 2015; Iannitti et al., 2014; Rahn et al., 2014) with a newer EAE-nPTX model that omitted PTX (Murphy et al., 2020). We report here that EAE-nPTX mice developed more, not less, severe motor dysfunction than EAE-PTX mice. Furthermore, EAE-PTX and EAE-nPTX mice displayed similar levels of mechanical, cold and heat hypersensitivity and spinal demyelination. Our results do not reject the use of PTX in standard EAE induction protocols. Rather, our more limited conclusion is that mice can develop EAE-associated behavioral and pathological signs of MS in the presence ***or absence*** of PTX. Removal of PTX eliminates potential confounds that could interfere with the assessment of pain mechanisms, sex-dependent differences, and the identification of therapeutic targets for MS-associated neuropathic pain. In summary, our findings demonstrate that PTX is not required to model pain-relevant features of EAE and suggest that its exclusion may improve the specificity of mechanistic studies by minimizing off-target effects on immune signaling.

A limitation of the comparison between the EAE-PTX and EAE-nPTX models is that they varied in MOG_35–55_ and MTB dose, which were slightly higher in the EAE-PTX model. The varied induction protocol in the PTX-free condition was assumed to be required to elicit demyelination and behavioral hypersensitivity (Murphy et al., 2020). To control for variable MOG_35–55_ and MTB dose and thus isolate the effects of PTX, we included the vEAE group (Table 1). The vEAE model did not induce signs of spinal cord demyelination, confirming that dose modification was necessary to induce disease in the absence of PTX. Surprisingly, the higher dose of MOG_35–55_ and MTB in vEAE (with PTX) was associated with a decrease in demyelination as compared to the EAE-PTX group. Future studies could more rigorously vary induction conditions to more precisely isolate and refine the interactions between levels of MOG, CFA, and PTX to induce maximal motor dysfunction, spinal cord pathology, and pain.

We found that EAE mice exhibited marked activation spinal dorsal horn microglia relative to naive controls, reflected by an increased IBA-1 immunoreactivity. This demonstrate a prolonged central neuroinflammatory response and so further validates the EAE-nPTX model as a relevant platform for investigating MS-associated neuropathic pain. This could be important because PTX directly stimulates the inflammatory response through cytokine production (Andreasen et al., 2009) and induction of lymphocytosis (Mu et al., 1994; Scanlon et al., 2019). PTX also ribosylates the α subunit of G_i/o_-proteins to prevent coupling with G-protein-coupled receptors (GPCRs) (Cassan et al., 2006; Mangmool & Kurose, 2011). G_i_-GPCR activation normally suppresses the adenylyl cyclase/cyclic AMP production promoted by G_s_-GPCRs (Godinho et al., 2015; Sunahara et al., 1996); however, PTX disrupts G_i_-mediated inhibition of adenylyl cyclase activity and leads to cyclic AMP accumulation (Mangmool & Kurose, 2011). PTX may alter EAE progression, particularly mechanisms involving inflammation and GPCRs, and this could confound the interpretation of research using traditional EAE models that include PTX. Omission of PTX from the EAE induction protocol may eliminate confounding effects of G_i_-GPCR uncoupling and thus permit more accurate investigation of GPCR-mediated mechanisms that underlie the central inflammatory response and pain in EAE.

Quantitative trait loci (QTL) mapping revealed sex-specific genetic influences in EAE models with and without PTX (Blankenhorn et al., 2000). QTL identifies DNA regions that regulate complex traits, such as EAE disease severity, spinal cord demyelination, and neuroinflammation. In EAE-PTX models, *eae9* governs disease susceptibility, mononuclear infiltration, and clinical severity. In EAE-nPTX models, *eae7* and *eae14* regulate disease severity and demyelination in both sexes, while *eae17*, *eae11*, and *eae18* exhibit sex-specific influences (Blankenhorn et al., 2000). We conclude that removal of PTX from the induction cocktail in MOG-based EAE models enhances its translational utility in C57BL/6 mice by eliminating PTX as a major confounding factor.

### 4.2. Potential sex differences

The inclusion of PTX in EAE models introduces variability that may confound assessments of sex-specific mechanisms underlying EAE-induced pain. We therefore conducted an exploratory sex-stratified analyses (Supplemental Fig. 1). Though limits in statistical power preclude definitive conclusions (each group contained three animals per sex), our preliminary findings suggest potential sex-dependent effects on motor dysfunction in EAE-PTX and EAE-nPTX models, with no consistent sex-based differences in pain sensitivity. Future studies with larger, sex-balanced cohorts will be necessary to rigorously examine the impact of sex on EAE-induced disease severity and pain.

### 4.3. Considerations of heat hypersensitivity in EAE and multiple sclerosis

When ambient or core body temperature rises, clinical symptoms of MS increase in 60–80 % of patients, a phenomenon attributed to reduced action potential conduction velocity (Christogianni et al., 2018; Davis et al., 2010; Olechowski et al., 2009; Rahn et al., 2014). Our preclinical studies revealed a very different form of heat hypersensitivity, represented by a decreased paw withdrawal threshold to cutaneous heat. This contrasts with some previous studies in EAE models, which reported reduced responsiveness to noxious heat (Olechowski et al., 2009) perhaps due to hindlimb paralysis, or no change (Rahn et al., 2014) perhaps due to older age. Albeit, the translational relevance of heat hypersensitivity remains unclear because it has not been demonstrated in clinical MS. For example, despite robust cutaneous hypersensitivity to mechanical and cold stimuli, as well as lower touch, pressure and cold detection thresholds (Osterberg & Boivie, 2010; Svendsen et al., 2005; Turri et al., 2018), quantitative sensory testing yielded no differences in heat hyperalgesia between patients with and without MS, or between MS patients who do or do not experience pain (Kakigi et al., 1992; Leocani et al., 2003; Osterberg & Boivie, 2010; Svendsen et al., 2005; Turri et al., 2018). In general however, these studies lacked rigor in terms of small sample sizes, inadequate sex stratification, incomplete reporting of MS subtype, insufficient evaluation of MS relapses, insufficient healthy controls, and a failure to distinguish between patients with or without pain. In a notable exception (Svendsen et al., 2005), 50 participants were included in each of MS-pain, MS-no pain, and control groups, with comparable numbers of men and women, and documented MS subtype. Rather than heat hypersensitivity, both MS groups exhibited the opposite: higher warm detection thresholds and diminished warm sensibility index as compared to controls (Svendsen et al., 2005). Similarly, Turri et al. observed increased heat detection thresholds in 28 MS patients compared to 20 healthy controls (Turri et al., 2018). To resolve these discrepancies, more rigorous investigations of heat threshold in EAE models and MS patients are needed.

### 4.4. Cortical demyelination is not present in PTX or nPTX models of EAE

Cortical demyelination is a hallmark of progressive MS. Although some studies have reported cortical demyelination in specific rat models (Storch et al., 2006), this has been difficult to replicate in mice. We found no evidence for cortical demyelination in mouse EAE-nPTX or EAE-PTX models, consistent with previous studies in C57BL/6 mice (Blankenhorn et al., 2000; Lagumersindez-Denis et al., 2017). In addition to lack of brain demyelination, murine EAE does not fully recapitulate other complexities of MS. First, unlike EAE, which requires immunization to induce disease, clinical manifestations of MS develop spontaneously, perhaps years after latent autoantigen sensitization (Constantinescu et al., 2011). Second, whereas EAE is predominantly associated with CD4 + T cell infiltration, clinical MS is associated not only with T cells but also chronic B cell activation and infiltration (Constantinescu et al., 2011; Gran et al., 2008). Albeit, given that cortical lesions are more strongly associated with cognitive and affective symptoms rather than sensory abnormalities, our study prioritized the assessment of spinal demyelination, which more directly relates to the somatosensory processing of noxious stimulation (Rivel et al., 2021).

### 4.5. Sensory neuron damage is present in EAE

It is widely thought that MS pathology is restricted to the central nervous system, and is not directly associated with peripheral neuropathy (Reich et al., 2018). This view that MS spares the peripheral nervous system was challenged by reports that some patients develop peripheral demyelinating neuropathy (Drake, 1987; Jende et al., 2017; Lassmann et al., 1981; Misawa et al., 2008; Saito et al., 1990). Whether this occurs in a significant proportion of the population is unclear. While some studies indicate a very small percentage of MS patients (Misawa et al., 2008; Suanprasert et al., 2019), others suggest a much greater prevalence: 1) Peripheral nerve lesions in every MS patient (Jende et al., 2017); 2) axonal polyneuropathy in electrophysiological studies in 14 of 18 patients (Lizarraga et al., 2021); 3) small-fiber neuropathy in leg biopsies of five out of eight MS patients with severe neuropathic pain (Rizvi et al., 2021); and 4) 45.5 % of MS patients had abnormal electrophysiologic nerve recordings without a clear correlation to neuropathic symptoms (Sarova-Pinhas et al., 1995). It is unclear from these studies whether peripheral involvement in MS is a secondary response to CNS damage, occurs in parallel with CNS degeneration, or is independent of the MS disease course. Additional work is necessary to establish the true prevalence of peripheral nerve pathology in MS and to determine its etiology.

In our study, the EAE-PTX model induced ATF3 expression in lumbar DRG neurons, in line with prior reports (Frezel et al., 2016; Yousuf et al., 2019). We observed ATF3 expression in both CFA-treated controls and EAE animals across all variants, indicating that DRG stress is not solely mediated by MOG_35–55_-induced T cell infiltration (Frezel et al., 2016). Notably, ATF3 levels were significantly higher in EAE-PTX mice than in CFA-PTX controls but were lower in EAE-nPTX mice relative to their CFA-nPTX controls. No differences were observed between vEAE and vCFA groups, and ATF3 expression was negligible in naïve animals. These findings suggest that adjuvant exposure, particularly in combination with PTX, contributes more strongly to peripheral neuronal stress than MOG immunization alone. Moreover, the overall extent of ATF3 expression was markedly lower than levels typically observed in models of peripheral nerve injury or neuropathic pain (Frezel et al., 2016; Hunt et al., 2012; Tsujino et al., 2000). Previous reports demonstrate that in EAE-PTX, ATF3 expression did not colocalize or associate with colony-stimulating factor 1 (CSF1) or neuropeptide-Y (NPY) expression, markers commonly upregulated following direct peripheral nerve damage (Frezel et al., 2016). Together, these results support the interpretation that a generalized stress response to systemic inflammation or PTX, rather than MOG_35–55_-driven neuropathology, contributes to ATF3 expression in lumbar DRG.

### 4.6. Future directions

While the current study highlights behavioral and pathological outcomes, a deeper mechanistic understanding will require additional approaches. The objective of the current study was to determine whether PTX is necessary to model MS-associated spinal demyelination and neuropathic pain, rather than to profile canonical markers of central neuroinflammation immune features such as GFAP, CCR2, or T-cell infiltration. To begin to fill this gap, ongoing work with the EAE-nPTX model is defining the contribution of microglial activation to MSNP.

## Supplementary Material

MMC1

## Figures and Tables

**Table 1:** Components of the EAE induction cocktail in each model.

MODEL	COMPONENT	Per side	Per Day	Total Amount

EAE-PTX				
	MOG_33–55_	75 ug	150 ug	**300 μg**
	MTB	0.15 mg	0.3 mg	**0.6 mg**
	IFA	50 ul	100 ul	**200 μl**
	PTX		200 ng	**400 ng**
EAE-nPTX	MOG_33–55_	50 ug	100 ug	**200 μg**
	MTB (total)			**0.5 mg**
	MTB (in CFA)*	0.025 mg	0.05 mg	
	MTB (added)	0.1 mg	0.2 mg	
	IFA (in CFA)*	25 μl	50 μl	**100 μl**
	PTX		0	**0**
vEAE	MOG_33–55_	75 ug	150 ug	**300 μg**
	MTB	.15 mg	0.3 mg	**0.6 mg**
	IFA	50 ul	100 ul	**200 μl**
	PTX		0	**0**
CFA-PTX	MOG_33–55_	75 ug	150 ug	**300 μg**
	MTB	0.15 mg	0.3 mg	**0.6 mg**
	IFA	50ul	100 ul	**200 μl**
	PTX		200 ng	**400 ng**
CFA-nPTX	MOG_33–55_	50 ug	100 ug	**200 μg**
	MTB (total)			**0.5 mg**
	MTB (in CFA)*	.025 mg	0.05 mg	
	MTB (added)	0.1 mg	0.2 mg	
	IFA (in CFA)*	25 μl	50 μl	**100 μl**
	PTX		0	**0**
vCFA	MOG_33–55_	75 ug	150 ug	**300 μg**
	MTB	0.15 mg	0.3 mg	**0.6 mg**
	IFA	50 ul	100 ul	**200 μl**
	PTX		0	**0**

Amounts of MOG, MTB, and IFA refer to that given at each of the two hind flanks, on each of two separate days. MOG_33-55_, Myelin Oligodendrocyte Glycoprotein peptide 35–55, MTB – *mycobacterium tuberculosis*, IFA – incomplete Freund’s adjuvant, PTX – pertussis toxin, *in the EAE-nPTX group, CFA (complete Freund’s adjuvant) provided the MTB and IFA at a concentration of 1 mg MTB per 1 ml IFA.

## Data Availability

The data that support the findings of this study are available from the corresponding author upon reasonable request.

## Appendix A. Supplementary data

Supplementary data to this article can be found online at https://doi.org/10.1016/j.brainres.2026.150162.

## Abbreviations:

MS: Multiple sclerosis
MSNP: multiple sclerosis associated neuropathic pain
EAE: experimental autoimmune encephalomyelitis
PTX: pertussis toxin
MOG: myelin oligodendrocyte glycoprotein
CFA: complete Freund’s adjuvant
