# Supplementary figures and images for: EAE models of neuropathic pain in multiple sclerosis do not require pertussis toxin
Source: Brain Res. Author manuscript; Available in PMC 2026 Aug 4. (PMC13436089; doi:10.1016/j.brainres.2026.150162)

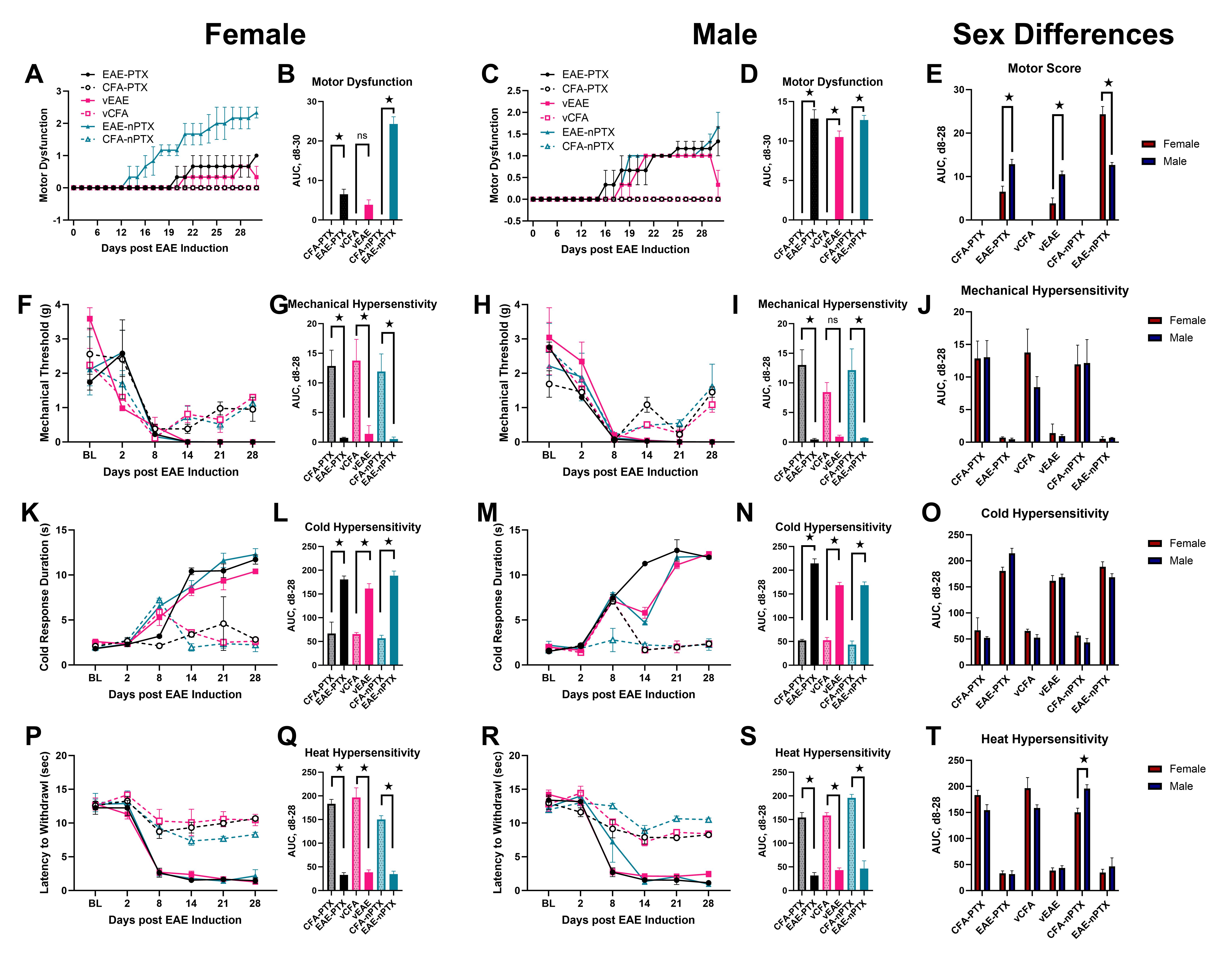

Supplement: MMC1 [file NIHMS2191186-supplement-MMC1.jpg]
